# ROS-induced epithelial-mesenchymal transition in mammary epithelial cells is mediated by NF-κB-dependent activation of Snail

**DOI:** 10.18632/oncotarget.1940

**Published:** 2014-05-01

**Authors:** Magdalena A. Cichon, Derek C. Radisky

**Affiliations:** ^1^ Mayo Clinic Cancer Center, 4500 San Pablo Road, Jacksonville, FL 32224, USA

**Keywords:** Epithelial-mesenchymal transition, matrix metalloproteinase-3, reactive oxygen species, nuclear factor-κB, Snail, breast cancer.

## Abstract

Epithelial-mesenchymal transition (EMT) is characterized by loss of cell-cell junctions, polarity and epithelial markers, and in turn, acquisition of mesenchymal features and motility. Changes associated with this developmental process have been extensively implicated in breast cancer progression and metastasis. Matrix metalloproteinases (MMPs) have been identified as specific inducers of EMT in mammary epithelial cells. MMP-3 induces EMT associated with malignant transformation via a pathway dependent upon production of reactive oxygen species (ROS). While the process by which exposure to MMP-3 leads to induction of ROS has been extensively studied, exactly how the MMP-3-induced ROS stimulate EMT remains unknown. Here, we used profiling methods to identify MMP-3-induced transcriptional alterations in mouse mammary epithelial cells, finding common overlap with changes mediated by nuclear factor-κB (NF-κB) and found in advanced breast cancer. In cultured cells, we found that Snail, an ROS-dependent key mediator of MMP-3-induced changes, is regulated by NF-κB in response to MMP-3. More specifically, we found MMP-3 to cause binding of p65 and cRel NF-κB subunits to the Snail promoter, leading to its transcription. Our results identify a specific pathway by which MMPs induce EMT and malignant characteristics, and provide insight into potential therapeutic approaches to target MMP-associated breast cancers.

## INTRODUCTION

Epithelial-mesenchymal transition (EMT) is a developmental reprogramming process in which polarized, immotile epithelial cells give rise to motile mesenchymal cells. In an orchestrated series of events, cell-cell and cell-extracellular matrix (ECM) interactions are altered to release epithelial cells from the tissue, the cytoskeletal structure and composition are reorganized to allow movement through the ECM, and a transcriptional program to maintain the mesenchymal phenotype is implemented [1-3]. Correct induction of the EMT program is critical in developmental processes including gastrulation and neural crest delamination [1, 4], however, inappropriate activation of EMT disturbs epithelial homeostasis and tissue integrity, contributing to many disease pathologies [5-7]. EMT processes identified in developmental studies have been found to be involved in key steps of tumor metastasis [8], and cancer-associated EMT also contributes to increased resistance to cell death, bypass of senescence, chemoresistance, immune system evasion and exhibition of cancer stem cell properties [7, 9-11]. Activation of EMT processes has been implicated in breast cancer disease pathology particularly. EMT is evident in transgenic mouse models of mammary carcinoma and human breast cancer patients [12, 13]. EMT markers and mediators have been particularly associated with basal and claudin-low breast cancer intrinsic subtypes [14-17]; patients who develop these subtypes of breast cancer are known to have poor clinical outcome [18]. Consequently, development of clinical approaches to target EMT for breast cancer patients is an important emerging objective, although identifying specific therapeutic targets and optimal patient populations remains a challenge [19].

Reactive oxygen species (ROS), produced as byproducts of metabolism or as a component of ROS-dependent signaling processes, have emerged as key mediators of cancer initiation and development [20]. Excess ROS can cause oncogenic mutations through direct reaction with DNA [20]. Additionally, ROS can induce metabolic reprogramming of cancer cells and surrounding stroma to facilitate tumor growth [21], and can act directly on cellular signaling pathways to drive the cancer phenotype through induction of EMT-associated pathways [22]. EMT-activating ROS can be endogenously produced as a result of general oxidative stress or activation of ROS-producing enzymes, or can be derived from stromal cells in the tumor microenvironment [23, 24]; the emerging paradigm is that the specificity of action of ROS for induction of EMT is not absolute, but is strictly dependent upon tissue type and cellular context [22].

Matrix metalloproteinases (MMPs), proteolytic enzymes that degrade and modify the ECM and also act directly on cell surface molecules, have been identified as another class of inducers that activate developmental EMT, as well as induce breast cancer-associated EMT [25]. MMP-3 (stromelysin-1), specifically, has been shown to cause EMT and to induce a premalignant phenotype in cultured mammary epithelial cells [26, 27], and to stimulate spontaneous tumor formation when expressed in mouse mammary glands [28]. We previously demonstrated that MMP-3 stimulates EMT in mammary epithelial cells via upregulation of Rac1b [29], an activated splice variant of Rac1 Rho GTPase [30-32], and further showed that MMP-3/Rac1b-induced EMT depended upon production of cellular ROS [29]. Recent studies have elucidated the role of extracellular cues from the ECM in controlling the generation and localization of Rac1b and subsequent production of ROS in breast cancer cells [33-35]. However, the intracellular mechanism by which MMP-3/Rac1b-induced ROS induce the EMT process has remained less well understood.

Here, we define the specific process by which MMP-3/Rac1b-induced ROS stimulate EMT in mammary epithelial cells. We show that MMP-3-induced EMT is dependent upon increased expression of the key EMT transcription factor Snail, that this increased expression is due to direct binding of the NF-κB subunits p65 and cRel bind to the promoter of Snail, and that this process is in turn dependent upon activation of p65/cRel by MMP-3-induced ROS. We also show that MMP-3/Rac1b-induced EMT activates transcriptional alterations characteristic of poor prognosis human breast cancer subtypes. Our results identify novel points for therapeutic targeting of EMT-associated processes in breast cancer progression.

## RESULTS

### Snail transcription factor is a necessary mediator of MMP-3-induced changes of gene expression and cell morphology

**Figure 1:**
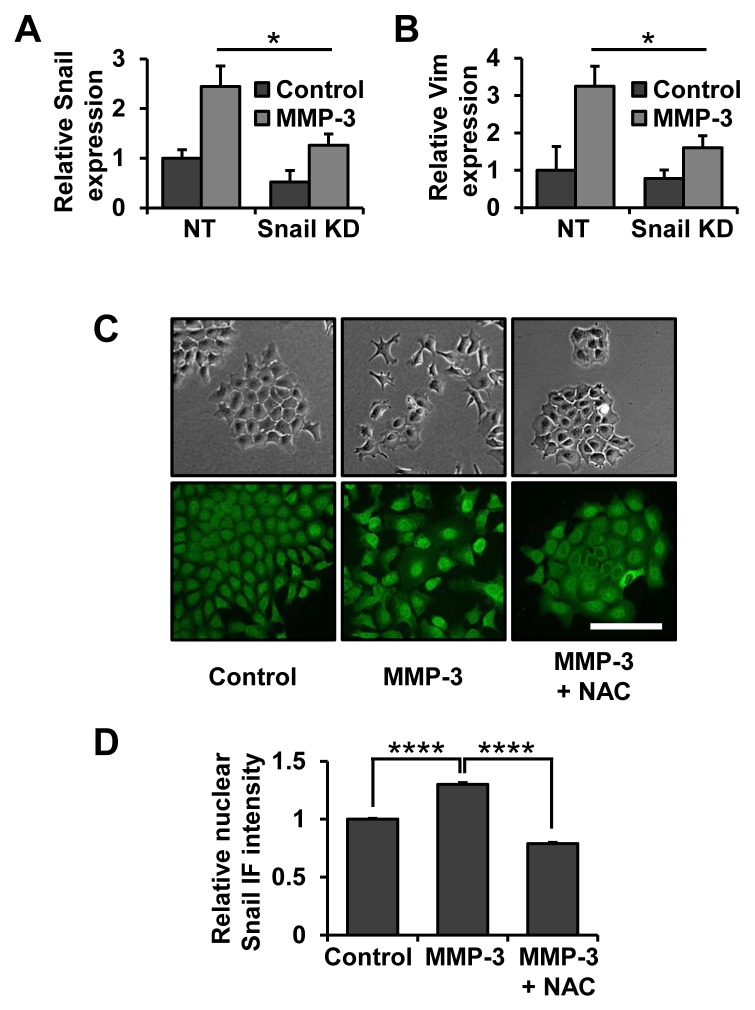
Snail is a necessary effector of MMP-3-induced alterations of gene expression and cell morphology **(A, B)** Non-target and Snail targeting shRNAs were delivered by lentiviral transduction, after which cells were exposed to MMP-3. The effect on Snail **(A)** and Vimentin **(B)** mRNA expression was assessed by RT-qPCR. Results are presented as relative quantification, normalized to GAPDH. **(C)** Phase contrast (top) and Snail immunofluorescence (bottom) images of cells exposed to MMP-3 or exposed to MMP-3 and treated with 10 mM N-acetyl-cysteine (NAC). Scale bar, 100 μm. **(D)** Quantification of nuclear Snail immunofluorescence intensity. At least 150 cells from at least five different fields were assessed for each condition. Error bars, SE. (* p<0.05; **** p<0.0001).

We have previously shown that MMP-3 treatment of mouse mammary epithelial cells leads to upregulation of the Snail transcription factor [29], a known key regulator of EMT, as well as cancer progression, invasion and metastasis [36, 37]. In order to further evaluate the role of Snail in MMP-3-induced EMT we used a lentiviral shRNA approach to decrease Snail expression levels. Exposure to MMP-3 led to increased Snail mRNA which was attenuated in cells with Snail knockdown (Fig. 1A). Snail knockdown was sufficient to inhibit MMP-3-induced vimentin upregulation (Fig. 1B), suggesting the role of Snail as a necessary mediator of this process. We further observed that morphology changes induced by MMP-3 were accompanied by increased levels of Snail specifically in the nuclei of the cells (Fig. 1C, D). These effects were blocked by treatment with N-acetyl-cysteine (NAC), showing their dependence on ROS (Fig. 1C, D).

### Gene expression profiling of cells treated with MMP-3 identifies pathways associated with ROS stimulation, NF-κB activation, and breast cancer progression

**Figure 2:**
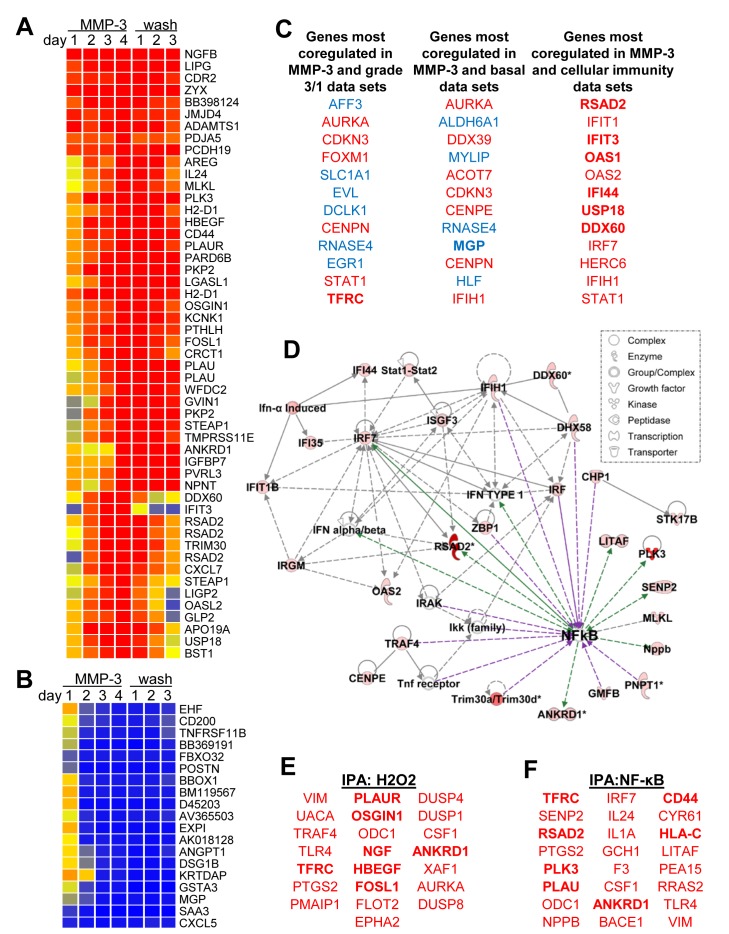
MMP-3 activates tumorigenic transcriptional profiles **(A-B)** Expression profiles of transcripts showing >10-fold upregulation **(A)** or downregulation **(B)** on day 4 in response to treatment with MMP-3. Cells were treated with media containing MMP-3 for four days and then with media without MMP-3 for three additional days. Samples were taken daily and analyzed for altered transcription as compared with a sample taken prior to treatment. **(C)** Top 10 coregulated transcripts in response to MMP-3 (using a dataset of transcripts showing fold change>3 on day 4) and published datasets comparing breast cancer grade 3 vs grade 1 samples (left), basal intrinsic subtype vs other subtypes (center), and cellular immune responses (right); upregulated transcripts are red; downregulated transcripts are blue. **(D)** Ingenuity Pathway Analysis (IPA) network of response to MMP-3 (intensity of red indicates degree of upregulation by MMP-3 on day 4). Direct interactions are indicated by solid lines, indirect by dashed lines; interactions that activate NF-κB are purple, those that are activated by NF-κB are green. **(E-F)** IPA also revealed enrichment for genes activated by hydrogen peroxide (E, overlap p-value 1.06E-07) and NF-κB (F, overlap p-value 2.89E-06).

In order to identify a potential mediator of MMP-3-induced Snail upregulation and EMT in mouse mammary epithelial cells, a microarray time course experiment was performed. SCp2 cells were exposed to MMP-3 for four days, and then allowed to recover in normal growth media for three additional days. Samples were taken daily and processed for total RNA; these were analyzed for transcriptional alterations relative to untreated samples. We found substantial and progressive transcriptional alterations associated with exposure to MMP-3 (Fig. 2A, B; annotated expression data in Supplemental Table S1). Consistent with previous studies implicating MMP-3 in EMT and in mammary branching morphogenesis, gene ontology analyses showed regulation of transcripts associated with cell adhesion (p=7.47E-05), cell-cell adhesion (p=2.25E-4), branching morphogenesis (p=3.32E-4), and response to wounding (p=3.3E-4; all categories with significant overlap listed in Supplemental Table S2).

We subjected the list of differentially expressed genes to a NextBio meta-analysis [38] (overlapping genesets with references provided in Supplemental Table S3), and found significant overlap with differentially expressed genes in eight datasets comparing profiles of breast cancer grade 3 vs grade 1 (Fig. 2C; Supplemental Fig. S1; Supplemental Table 4) and with seven datasets comparing profiles of breast cancer of the basal intrinsic subtype vs normal breast tissue or with breast cancers of other subtypes (Fig. 2C; Supplemental Fig. S2; Supplemental Table S5). The NextBio meta-analysis also showed significant overlap with differentially expressed genes in six datasets comparing cells with activated immune responses (Fig. 2C; Supplemental Fig. S3; Supplemental Table S6), suggesting that MMP-3 was activating immune-related pathways. Using Ingenuity Pathway Analysis (IPA) of the differentially expressed genes, we identified an interaction network that centered on the transcription factor and immune response mediator NF-κB (Fig. 2D), as well as significant enrichment of genes predicted to be regulated by hydrogen peroxide (H2O2; Fig. 2E), which we have shown to be an effective inducer of MMP-3-activated pathways [29, 33, 39] and which is an inducer of NF-κB, as well as genes predicted to be directly regulated by NF-κB (Fig. 2F) [40]. IPA analysis of the differentially expressed genes also identified an EMT-associated interaction network linking MMPs, ECM molecules, growth factors, and oncogenes (Supplemental Fig. S4).

### MMP-3 responsive region of the Snail promoter contains an NF-κB binding site

**Figure 3:**
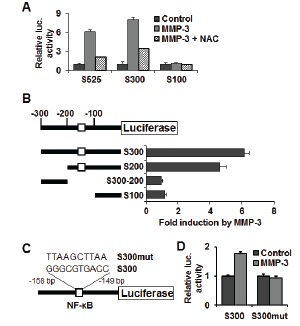
MMP-3 responsive region of the Snail promoter contains an NF-κB binding site **(A)** Luciferase reporters under control of mouse Snail promoter region −525, −300 or −100 bp upstream of Snail translation start site, respectively, reveal MMP-3-responsive element localized in the −300/−100 bp region. **(B)** Further promoter reporter deletions identify the MMP-3 responsive region to be localized −200/−100 bp upstream of the Snail translation start site. Schematic representation of the mouse Snail promoter region (top) and deletion mutants tested (bottom), showing the position of potential regulatory elements. **(C)** Diagram showing mutation of the candidate NF-κB binding site in the Snail promoter reporter. **(D)** Mutation of the candidate NF-κB binding site in the S300 reporter inhibits its induction by MMP-3. (A), (B), (D) Error bars, SE of triplicates.

In order to determine the MMP-3 responsive region of the Snail promoter we utilized luciferase reporter constructs under control of promoter regions 525, 300 and 100 bp upstream of the Snail transcription start site (courtesy A. Cano [41]) (Fig. 3A). MMP-3 treatment activated the S525 and S300 Snail promoter reporters but not the S100, indicating that the MMP-3-responsive region is localized between 100 and 300 bp upstream of the Snail transcription start site. NAC blocked MMP-3-induction of the Snail promoter reporter constructs, showing that this process also is ROS dependent. Deletional mutagenesis within the 300 bp region of the S300 Snail promoter reporter allowed us to further minimize the MMP-3 responsive domain to the region between 100 and 200 bp upstream of the Snail transcription start site (Fig. 3B). Using the TFSEARCH tool to search the TRANSFAC database [42], we identified a potential binding site for NF-κB with homology to NFKAPPAB_01 (GGGAMTTYCC, score 69.5) and CREL_01 (SGGRNWTTCC, score 69.8). We determined that mutation of this site in the S300 luciferase reporter resulted in loss of activation of the reporter by MMP-3 (Fig. 3D).

### MMP-3-induced Snail upregulation occurs via ROS-dependent NF-κB activation

**Figure 4:**
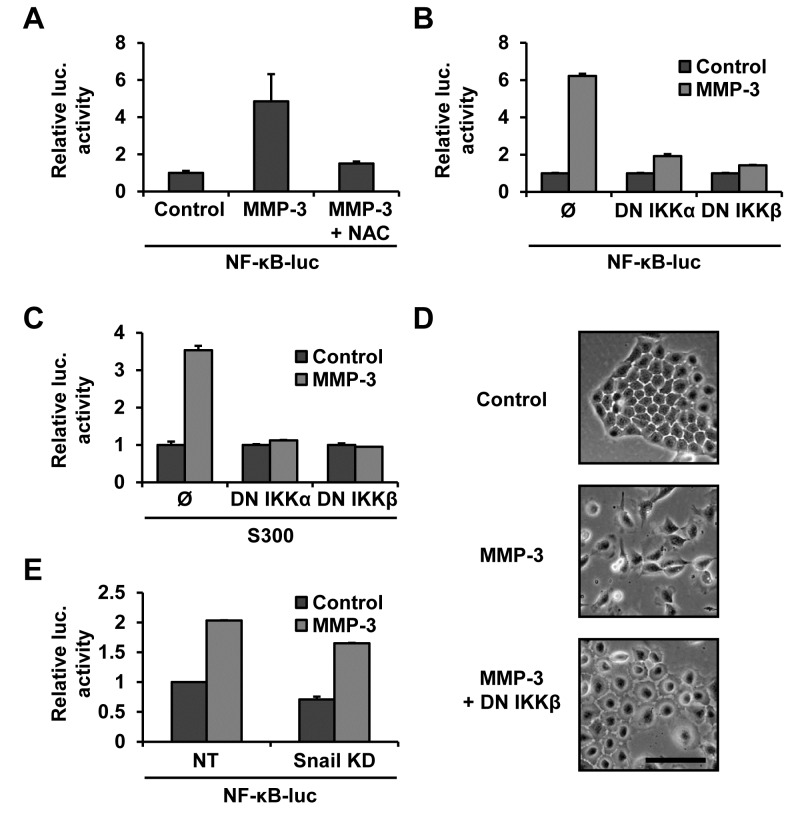
MMP-3-induced Snail upregulation is dependent on NF-κB **(A)** MMP-3 activates NF-κB in an ROS dependent manner. Cells were transfected with an NF-κB luciferase reporter and exposed to MMP-3 alone or MMP-3 and 10mM NAC. **(B)** Co-transfection with dominant negative IKKα or IKKβ, super-repressors of the NF-κB pathway, blocks MMP-3-induced activation of NF-κB luciferase. **(C)** Snail promoter activation by MMP-3 is NF-κB dependent. Co-transfection with dominant negative IKKs inhibited MMP-3-induced Snail promoter reporter activation. Luciferase activity was normalized to protein concentration. **(D)** Phase contrast images of cells transfected with a control vector or dominant negative IKKβ and exposed to MMP-3. Scale bar, 100 μm. **(E)** Snail knockdown does not affect MMP-3-induced NF-κB activation. Non-target and Snail targeting shRNAs were delivered by lentiviral transduction, after which cells were transfected with NF-κB luciferase, followed by exposure to MMP-3. Luciferase activity was normalized to untreated samples for each transduction condition. Error bars, SE.

To test the involvement of NF-κB in the MMP-3-induced Snail upregulation we used NF-κB responsive luciferase reporter construct and genetic inhibitors of the NF-κB activation pathway (courtesy M. Karin [43, 44]). We found that MMP-3 increased NF-κB activity and that this was ROS dependent as it could be blocked by NAC treatment (Fig. 4A). We also found that co-transfection with the dominant negative IKKα and IKKβ abrogated this activation (Fig. 4B). Each of the dominant negative super repressors was also sufficient to block Snail promoter activation by MMP-3, demonstrating that the Snail promoter activation by MMP-3 is NF-κB dependent (Fig. 4C). MMP-3-induced cell morphology changes were also inhibited by inhibiting the NF-κB pathway (Fig. 4D). Finally, we found that cells in which Snail had been silenced (Fig. 1A) showed no diminishment of NF-κB activation by MMP-3, confirming linearity of the pathway (Fig. 4E). To obtain further support for a role of NF-κB activation in response to treatment with MMP-3, we used luciferase promoters reporters specific to seven other MMP-3-regulated genes, and found that co-transfection with the dominant negative IKKβ construct blocked MMP-3-induced gene induction for these constructs as well (Supplemental Fig. S5).

### p65 and cRel subunits of NF-κB bind to the Snail promoter in response to MMP-3

**Figure 5:**
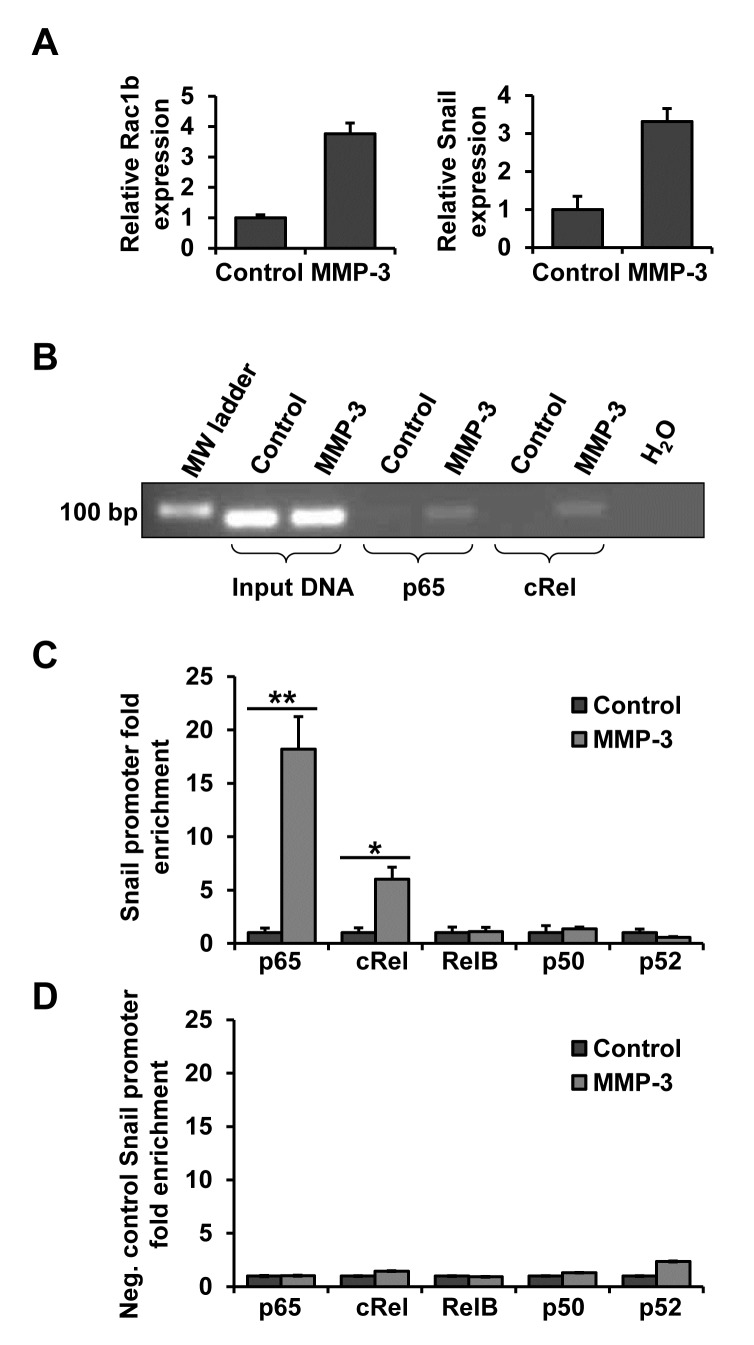
NF-κB subunits p65 and cRel bind to the Snail promoter in response to MMP-3 DNA from control and MMP-3-exposed cells was subjected to chromatin immunoprecipitation (ChIP) using antibodies against five NF-κB subunits. **(A)** MMP-3 response in the samples used for ChIP showed substantial increases in Rac1b (left) and Snail (right). **(B)** DNA pulled down with p65 and cRel was analyzed by semi-quantitative PCR. **(C, D)** DNA pulled down with antibodies against all five NF-κB subunits was analyzed by RT-qPCR, using custom primers and probe flanking the candidate NF-κB binding site in the proximal Snail promoter region **(C)** or negative control custom primers and probe flanking a potential NF-κB binding site in the distal Snail promoter region **(D)**. For each antibody the amount of chromatin pulled down from cells exposed to MMP-3 is presented as fold enrichment over control samples. Error bars, SE. (* p<0.05; ** p<0.01).

As NF-κB is a family of 5 subunits which can homo- and heterodimerize we performed chromatin immunoprecipitation (ChIP) in order to determine whether and which NF-κB subunits bind to the Snail promoter in response to MMP-3. After confirming in parallel samples that MMP-3 led to increased Rac1b and Snail transcript levels (Fig. 5A), lysates from control and MMP-3 exposed cells were subjected to ChIP with antibodies to the five NF-κB subunits. Binding to the Snail promoter was analyzed by semi-quantitiative PCR (Fig. 5B), as well as RT-qPCR, with primers flanking the NF-κB binding site of the Snail promoter region (Fig. 5C). MMP-3 treatment increased abundance of the Snail promoter region containing the NF-κB binding site in the samples that had been pulled down with p65 and cRel antibodies, thus demonstrating that MMP-3-induced Snail upregulation is mediated by binding of the NF-κB heterodimer comprised of p65 and cRel to the Snail promoter. To confirm specificity of the MMP-3-induced p65/cRel binding at this site on the Snail promoter, we evaluated an NF-κB/cRel binding site at −1.6 kb (−1593/−1607; AAGGGAGCTTCCTGG). Custom Taqman RT-qPCR primers and probe were designed to span this specific region and used as a negative control for ChIP, testing chromatin from the same experiment as the one with the specific probe of interest showed no enrichment in either of the samples (Fig. 5D).

## DISCUSSION

The process of tumor-associated EMT is known to contribute to cancer progression and metastasis [7, 19] and ROS has been extensively implicated as an inducer of EMT [22]. Here, we provide important new insight into MMP-induced, ROS-dependent EMT in breast cancer [29]. We found that MMP-3-regulated genes overlapped with datasets of higher breast cancer grades and poorer prognosis breast cancer subtypes. Further analysis pointed towards regulation of immune pathways and genes controlled by ROS, and finally revealed a signaling network centered around NF-κB. We identified the region of the Snail promoter activated by MMP-3 induced ROS, finding it to contain a binding site for NF-κB, a transcription factor that has also been previously implicated in EMT and in cancer development and progression [45-47]. We further found that this NF-κB-responsive element was necessary for MMP-3-induced ROS activation of Snail promoter activity, and we defined the specific NF-κB subunits involved as p65 and cRel. We extended this paradigm, identifying several other MMP-3/ROS-induced genes as dependent on NF-κB. Defining NF-κB-induced Snail activation as the critical step in MMP/ROS-induced EMT reveals new avenues of potential therapeutic intervention for breast cancer patients in whom these pathways are activated.

Snail has been implicated in breast cancer metastasis and associated with higher tumor grade, disease recurrence, poor prognosis and decreased survival [36, 48-50]. We found Snail to be a key mediator in MMP-3/ROS-induced EMT and gene expression changes associated with this process overlap with datasets from cells with activated immune responses. Snail has been most extensively studied in the context of inflammation-induced EMT. Its AKT/GSK-3β-mediated stabilization has been demonstrated to be necessary for TNF-α-induced EMT in colorectal cancer cells [51]. Similar findings were shown in TNF-α-induced breast cancer associated EMT and metastasis, where those processes were reliant on NF-κB-dependent Snail stabilization [52]; such stromal activation is a key feature of breast cancer progression. In other work, TGFβ-induced Snail expression in hepatocytes was found to be dependent on p50/p65 binding at −162 bp [53], and p65 was able to activate Snail promoter in colon cancer cells via a region between −194 and −78 bp [54]; these studies indicate that the subunit specificity is likely cell type and stimulus dependent. Our findings show MMP-3/ROS-induced and NF-κB-mediated Snail transcription as a functional link between increased MMP expression in breast cancers and progression to metastasis that could be targeted for therapeutic response.

We also found our dataset of MMP-3-induced transcriptional responses to overlap with transcriptional profiles of higher grade and more aggressive breast cancer subtypes. While MMPs have been identified as potential mediators of breast cancer development and progression [25], defining a specific role for individual MMPs in specific subtypes of human breast cancer has been elusive. It is potentially relevant that MMP-3, which is one of the central mediators of postlactational involution [55], a process associated with increased cancer risk in premenopausal women [56, 57], induces in cultured mammary epithelial cells transcriptional responses similar to those found in aggressive, basal, pregnancy-associated breast cancers [56, 58].

Here, we inhibited MMP-3-induced ROS and downstream effects using NAC, however, it should be noted that this reagent has additional effects. For example NAC has been shown to inhibit mammalian target of rapamycin (mTOR; [59]), possibly by decreasing ROS and consequently inhibiting the positive feedback between mTOR and ROS [60]. As mTOR has been implicated in EMT [61, 62], it is a possibility that some of the changes induced by MMP-3-induced ROS could be associated with the mTOR pathway. This possibility is currently under investigation.

MMP-3-induced production of cellular ROS and consequent EMT in mammary epithelial cells has been found to involve specific generation of Rac1b, an activated, alternative splice isoform of Rac1 [29, 63]. Rac1b has also been found to play a role in NF-κB-dependent activation of colorectal cancer cells [30, 64, 65] and development of lung cancer [11, 66, 67]. Production of cellular ROS downstream of Rac1b [29] is dependent on the environmental context: cells must be able to undergo morphological alterations [39] and respond to appropriate ECM cues [33-35]. In the context of stiff ECM, Rac1b localizes to cell membrane where it can facilitate assembly of NADPH oxidase which consequently catalyzes production of ROS. Microenvironment stiffness as a regulator of the MMP-3-induced EMT points towards possible specific relevance of this pathway in stiffer, more collagen-rich environments such as cancer-associated fibrosis, which are more common in highly aggressive, basal subtype cancers [68]. Significantly, in the present study we found overlap of the geneset induced by MMP-3/ROS signaling in mammary epithelial cells with multiple datasets from other studies comparing the basal intrinsic subtype of breast cancer with other, less aggressive, subtypes. This suggests that the EMT mechanisms studied here, and their implications for novel points of therapeutic intervention, may be particularly relevant for basal breast cancer.

## CONCLUSIONS

Cancer-associated EMT is known to contribute to tumor progression, increased invasiveness and metastasis, resistance to therapies, and generation of cell populations with stem cell-like characteristics [19, 69, 70], and has been implicated in progression and metastasis of breast cancer specifically [12, 14, 17]. The presented study unifies several known EMT mediators, showing that MMP-3/Rac1b/ROS induction of EMT in mammary epithelial cells proceeds via activation of NF-κB signaling and direct transcriptional activation of Snail, and thus providing new insights into the mechanism of EMT induction in the context of breast tumor progression. Specifically, our results implicate p65/cRel as mediators in MMP-3-induced, ROS-mediated process of EMT. Given that we have found this process to be most commonly activated in basal-like cancers, these findings indicate a novel avenue for therapeutic intervention with ROS-targeting and/or heterodimer-specific inhibitors of NF-κB.

## MATERIALS AND METHODS

### Cell culture

Culture of SCp2 nontumorigenic mouse mammary epithelial cells and induction by MMP-3 was performed as described previously [29, 33]. Cell culture medium consisted of DMEM/F12 (Invitrogen) supplemented with 2% Tet System Approved FBS (Clontech), 0.4 mg/ml G418 sulfate (Cellgro), 50 μg/ml gentamicin (Invitrogen), 5 μg/ml human recombinant insulin (Invitrogen). N-acetyl cysteine (NAC) (Sigma) treatment was performed at a concentration of 10 mM. For luciferase assays, cell lysates were prepared with Reporter Gene Assay lysis buffer (Roche) and analyzed using Dual-Luciferase Reporter Assay System (Promega) or with Luciferase Assay System (Promega) in a Veritas Microplate Luminometer (Turner Biosystems), according to the manufacturer's instructions. The *Firefly* luciferase activity was normalized to either *Renilla* luciferase activity or protein concentration determined in a BCA assay (Thermo Scientific). All samples were assayed in triplicates.

### Transcriptional analysis

Samples of SCp2 cells were exposed to MMP-3 for four days, washed and then grown in normal media for three additional days, with samples taken daily. Isolated total RNA was assessed with Affymetrix mouse 430_2 gene expression chips and processed and analyzed using Genespring GX utilizing previously described methods [11, 71, 72]; transcriptional profiles have been deposited in Gene Expression Omnibus (GEO accession number: GSE56909). Meta-analysis was performed using Nextbio [38]. Ingenuity Pathway Analysis (IPA) was performed using the web interface (www.ingenuity.com) to build gene interactions associated with response to MMP-3. Transcription factor analysis was performed using the TFSEARCH tool (http://www.rwcp.or.jp/lab/pdappl/papia.html), which searches the TRANSFAC database [42].

### Constructs, transfection and lentiviral transduction

Luciferase reporter constructs controlled by different regions of the mouse Snail promoter were obtained from Dr. Amparo Cano [41]. Mutagenesis of the Snail promoter reporter was performed using Quick Change mutagenesis kit (Stratagene) and the S300 construct as template using specific primers: for S200, 5'-GCCAATGGCTGGCGGGGATCCAGA-CATGATAAG-3' and 5'-CTTATCATGTCTGGATCC CCGCCGCC AGCCATTGGC-3'; for S300-200, 5'-GTCGACCCGGGTACCCGGCGCGGCCGGAG-3' and 5'-CTCCGGCCGCCGCGCCGGGTACCCGGGTC GAC-3'; for mutagenesis of the putative NF-κB binding site, 5'-GGGGGCGTGACCGTACTGTTTTAAG CTTAACTTTGTCAAGGC-3'; 5'-GCCTTGACAAAGT TAAGCTTAAA ACAGTACGGTCACGCCCCC-3'. NF-κB responsive luciferase reporter construct and genetic inhibitors of the NF-κB activation pathway, dominant-negative IKKα and IKKβ mutants were obtained from Dr. Michael Karin [43, 44]. In addition following luciferase promoter reporters were used: KRT16 [73], CXCL1 [74], CEBPD [75], OPG [76], GSTA3 [77], SAA3 [78], CYR61 [79]. Transfections were done with 2 μg of a given construct per well in a 6-well plate. The cells were co-transfected with 100 ng pRL-CMV (Promega) *Renilla* luciferase construct for normalization. Cells stably expressing NF-κB-luc were obtained by co-transfection with pcDNA3 hygromycin resistance construct and 150 μg/ml hygromycin (Calbiochem) selection. Transfections were performed using Lipofectamine 2000 reagent (Invitrogen), according to the manufacturer's instructions. Snail knockdown was obtained by lentiviral transduction with Mission shRNA NM_011427.2-1436s21c1, targeting sequence 5'-ATGTGTCTCCCAGAACTATTT-3' (Sigma). Non-target shRNA (SHC216, Sigma) 5'-CCGGGCGCGATAGCGCTAATAATTTCTCGAGAAATTATTAGCGCTATCG-CGCTTTTT-3' was used as a control.

### Real-time quantitative PCR (RT-qPCR)

RNA was isolated using Trizol reagent (Invitrogen) according to the manufacturer's instructions. cDNA was synthesized with Multiscribe reverse transcriptase (Applied Biosystems). Gene expression levels were assayed by real-time quantitative PCR (RT-qPCR) using 7900 HT Fast Real-Time PCR system and TaqMan probes for specific genes (Snai1 Mm00441533_g1, Vimentin Mm00449201_m1) (Applied Biosystems). For Rac1b, custom primers and probe were used: forward, 5'-TGGACAAGAAGATTATGACAGATTGC-3'; reverse, 5'-CCCTGGAGGGTCTATCTTTACCA-3'; probe, 5'-CCGCAGACAGTTGGAGA-3'. Samples were assayed in triplicates for each probe. GAPDH custom primers and probe were used as endogenous control to normalize expression levels: forward, 5'- GTGTCCGTCGTGGATCTGA-3'; reverse 5'- GCTTCACCACCTTCTTGATGTCAT-3'; probe, 5'- CTTGGCAGGTTTCTCC-3'. Relative quantification analysis was performed using RQ Manager 1.2.1 software (Applied Biosystems).

### Snail immunofluorescence and analysis

Cells were fixed and permeabilized with ice cold methanol at −20°C for 10 min, and blocked in blocking buffer (0.2% TritonX, 1% BSA, 10% goat serum in PBS) at 4°C overnight. Snail immunofluorescence was performed using anti-Snail antibody (ab85931, Abcam), 1:500, followed by Alexa Fluor 488 goat anti-rabbit IgG (Invitrogen), 1:1000, each incubation done at room temperature for 1 hour. Nuclei were stained with Hoechst (Invitrogen), 1:10 000. Pictures were taken using Olympus IX71 microscope (Olympus) with QuantiFire XI camera (Optronics). Nuclear Snail staining intensity was analyzed using ImageJ [80].

### Chromatin Immunoprecipitation

Chromatin immunoprecipitation (ChIP) was performed with ChIP-IT Express Enzymatic kit (Active Motif). The cells were fixed with 1% formaldehyde in DMEM/F12 (Invitrogen) at room temperature for 10 min to cross-link and preserve protein/DNA interactions, lyzed and the chromatin was sheared into fragments by enzymatic digestion at 37°C for 10 min. The sheared chromatin was incubated on a rolling shaker overnight at 4°C with antibodies against the five NF-κB subunits (2 μg per reaction) and Protein G-coated magnetic beads. The following rabbit polyclonal antibodies were used: NF-κB p50 (H-119): sc-7178; NF-κB p52 (K-27): sc-298; NF-κB p65 (C-20): sc-372; RelB (C-19): sc-226; c-Rel (C): sc-71 (Santa Cruz). Through the use of Protein G beads, antibody-bound protein/DNA complexes were precipitated. The captured chromatin was then eluted, cross-linking was reversed at 94°C for 15 min, and proteins were removed by treatment with Proteinase K at 37°C for 1 hour. The DNA was analyzed using an NF-κB Snail promoter-specific custom TaqMan assay: forward primer, 5'-CTGTCAG GTGACCGTTCATTGA-3'; reverse primer, 5'-GATTCGAATACTAAAGGGAGGTGTGA-3'; reporter, 5'-TCCCCACCTCCTTTC-3', and a control TaqMan assay designed to amplify a region 1.6 kb upstream of the NF-κB binding site: forward primer, 5'-TGGCAGTCGGCCTAGGAA-3'; reverse primer, 5'-ACGGGTGACAGGTGACTCT-3'; reporter, 5'-CCCAGGAAGCTCCC-3' (Applied Biosystems). Samples were assessed in sets of 6 by RT-qPCR and data was analyzed using 2^-ΔΔCt method. Ct values of samples pulled down with specific antibodies were normalized to Ct of input samples. The samples were also amplified by conventional PCR, using the above NF-κB Snail promoter-specific forward and reverse primers and Platinium Taq DNA Polymerase (Invitrogen).

### Statistical analysis

Statistical analyses were performed using GraphPad software. Statistical significance was determined by student's t-test.

## SUPPLEMENTARY TABLES, FIGURES AND REFERENCES















## ABBREVIATIONS

ChIP: chromatin immunoprecipitation
ECM: extracellular matrix
EMT: epithelial-mesenchymal transition
IPA: Ingenuity Pathway Analysis
MMPs: matrix metalloproteinases
mTOR: mammalian target of rapamycin
NAC: N-acetyl cysteine
NF-κB: nuclear factor-κB
ROS: reactive oxygen species
