# Supplementary material for: ROS-induced epithelial-mesenchymal transition in mammary epithelial cells is mediated by NF-κB-dependent activation of Snail
Source: Oncotarget. 2014 May 1;5(9):2827–38. doi: 10.18632/oncotarget.1940 (PMC4058048; doi:10.18632/oncotarget.1940)
Supplement: Supplementary file 3 [file oncotarget-05-2827-s003.pdf]

Supplemental Table S3: Overlapping genesets identified through meta-analysis

Overlapping genesets	p-value	reference
**Grade 3 vs Grade 1**		
SBR grade 3 vs 1	1.3E-12	[1]
SBR grade 3 vs 1	2.0E-12	[2]
BR grade 3 vs 1	5.7E-12	[3]
Elston grade 3 vs 1	3.7E-12	[4]
Elson grade 3 vs 1	1.7E-14	[5]
Elston grade 3 vs 1	1.2E-13	[6]
Grade 3 vs 1	1.9E-12	[7]
Pathological grade 3 vs 1	3.2E-14	[8]
**Basal**		
Basal-like vs normal	9.5E-10	[9]
Basal IHC vs normal	1.1E-07	[10]
Basal molecular vs normal	1.0E-16	[1]
Basal vs normal breast tissue	3.7E-16	[2]
Basal vs "normal-like"	1.6E-13	[4]
Luminal vs Basal B	3.9E-11	[11]
Basal B vs Basal A	3.4E-01	[11]
**Interferon response**		
A549+IFNα	4.3E-10	[12]
Calu3+IFNα	6.6E-22	[13]
Bronchial epithelia+IFNβ	5.7E-12	[14]
Hepatocytes+IFNα	7.4E-18	[15]
Keratinocytes+IFNα	1.8E-12	[16]
Melanoma+IFNα	7.8E-12	[17]

SUPPLEMENTAL TABLE S3 REFERENCES

1. Sircoulomb F, Bekhouche I, Finetti P, Adelaide J, Ben Hamida A, Bonansea J, Raynaud S, Innocenti C, Charafe-Jauffret E, Tarpin C, Ben Ayed F, Viens P, Jacquemier J, Bertucci F, Birnbaum D and Chaffanet M. Genome profiling of ERBB2-amplified breast cancers. BMC Cancer. 2010; 10:539.
2. Sabatier R, Finetti P, Adelaide J, Guille A, Borg JP, Chaffanet M, Lane L, Birnbaum D and Bertucci F. Down-regulation of ECRG4, a candidate tumor suppressor gene, in human breast cancer. PLoS One. 2011; 6(11):e27656.
3. Lu X, Wang ZC, Iglehart JD, Zhang X and Richardson AL. Predicting features of breast cancer with gene expression patterns. Breast Cancer Res Treat. 2008; 108(2):191-201.
4. Pawitan Y, Bjohle J, Amler L, Borg AL, Egyhazi S, Hall P, Han X, Holmberg L, Huang F, Klaar S, Liu ET, Miller L, Nordgren H, Ploner A, Sandelin K, Shaw PM, et al. Gene expression profiling spares early breast cancer patients from adjuvant therapy: derived and validated in two population-based cohorts. Breast Cancer Res. 2005; 7(6):R953-964.
5. Ivshina AV, George J, Senko O, Mow B, Putti TC, Smeds J, Lindahl T, Pawitan Y, Hall P, Nordgren H, Wong JE, Liu ET, Bergh J, Kuznetsov VA and Miller LD. Genetic reclassification of histologic grade delineates new clinical subtypes of breast cancer. Cancer Res. 2006; 66(21):10292-10301.
6. Miller LD, Smeds J, George J, Vega VB, Vergara L, Ploner A, Pawitan Y, Hall P, Klaar S, Liu ET and Bergh J. An expression signature for p53 status in human breast cancer predicts mutation status, transcriptional effects, and patient survival. Proc Natl Acad Sci U S A. 2005; 102(38):13550-13555.
7. Silver DP, Richardson AL, Eklund AC, Wang ZC, Szallasi Z, Li Q, Juul N, Leong CO, Calogrias D, Buraimoh A, Fatima A, Gelman RS, Ryan PD, Tung NM, De Nicolo A, Ganesan S, et al. Efficacy of neoadjuvant Cisplatin in triple-negative breast cancer. J Clin Oncol. 2010; 28(7):1145-1153.
8. Oncology) TIGCITepEPf. The International Genomics Consortium (IGC). The expO project (Expression Project for Oncology).
9. Parker JS, Mullins M, Cheang MC, Leung S, Voduc D, Vickery T, Davies S, Fauron C, He X, Hu Z, Quackenbush JF, Stijleman IJ, Palazzo J, Marron JS, Nobel AB, Mardis E, et al. Supervised risk predictor of breast cancer based on intrinsic subtypes. J Clin Oncol. 2009; 27(8):1160-1167.
10. Dedeurwaerder S, Desmedt C, Calonne E, Singhal SK, Haibe-Kains B, Defrance M, Michiels S, Volkmar M, Deplus R, Luciani J, Lallemand F, Larsimont D, Toussaint J, Haussy S, Rothe F, Rouas G, et al. DNA methylation profiling reveals a predominant immune component in breast cancers. EMBO Mol Med. 2011; 3(12):726-741.
11. Neve RM, Chin K, Fridlyand J, Yeh J, Baehner FL, Fevr T, Clark L, Bayani N, Coppe JP, Tong F, Speed T, Spellman PT, DeVries S, Lapuk A, Wang NJ, Kuo WL, et al. A collection of breast cancer cell lines for the study of functionally distinct cancer subtypes. Cancer Cell. 2006; 10(6):515-527.
12. Sanda C, Weitzel P, Tsukahara T, Schaley J, Edenberg HJ, Stephens MA, McClintick JN, Blatt LM, Li L, Brodsky L and Taylor MW. Differential gene induction by type I and type II interferons and their combination. J Interferon Cytokine Res. 2006; 26(7):462-472.
13. Li C, Bankhead A, 3rd, Eisfeld AJ, Hatta Y, Jeng S, Chang JH, Aicher LD, Proll S, Ellis AL, Law GL, Waters KM, Neumann G, Katze MG, McWeeney S and Kawaoka Y. Host

regulatory network response to infection with highly pathogenic H5N1 avian influenza virus. J Virol. 2011; 85(21):10955-10967.
14. Shapira SD, Gat-Viks I, Shum BO, Dricot A, de Grace MM, Wu L, Gupta PB, Hao T, Silver SJ, Root DE, Hill DE, Regev A and Hacohen N. A physical and regulatory map of host-influenza interactions reveals pathways in H1N1 infection. Cell. 2009; 139(7):1255-1267.
15. Thomas E, Gonzalez VD, Li Q, Modi AA, Chen W, Noureddin M, Rotman Y and Liang TJ. HCV infection induces a unique hepatic innate immune response associated with robust production of type III interferons. Gastroenterology. 2012; 142(4):978-988.
16. Swindell WR, Xing X, Stuart PE, Chen CS, Aphale A, Nair RP, Voorhees JJ, Elder JT, Johnston A and Gudjonsson JE. Heterogeneity of inflammatory and cytokine networks in chronic plaque psoriasis. PLoS One. 2012; 7(3):e34594.
17. Kholmanskikh O, van Baren N, Brasseur F, Ottaviani S, Vanacker J, Arts N, van der Bruggen P, Coulie P and De Plaen E. Interleukins 1alpha and 1beta secreted by some melanoma cell lines strongly reduce expression of MITF-M and melanocyte differentiation antigens. Int J Cancer. 2010; 127(7):1625-1636.
