# Supplementary material for: ROS-induced epithelial-mesenchymal transition in mammary epithelial cells is mediated by NF-κB-dependent activation of Snail
Source: Oncotarget. 2014 May 1;5(9):2827–38. doi: 10.18632/oncotarget.1940 (PMC4058048; doi:10.18632/oncotarget.1940)
Supplement: Supplementary file 7 [file oncotarget-05-2827-s007.pdf]

ROS-induced epithelial-mesenchymal transition in mammary epithelial cells is mediated by NF-κB-dependent activation of Snail

Supplementary Material

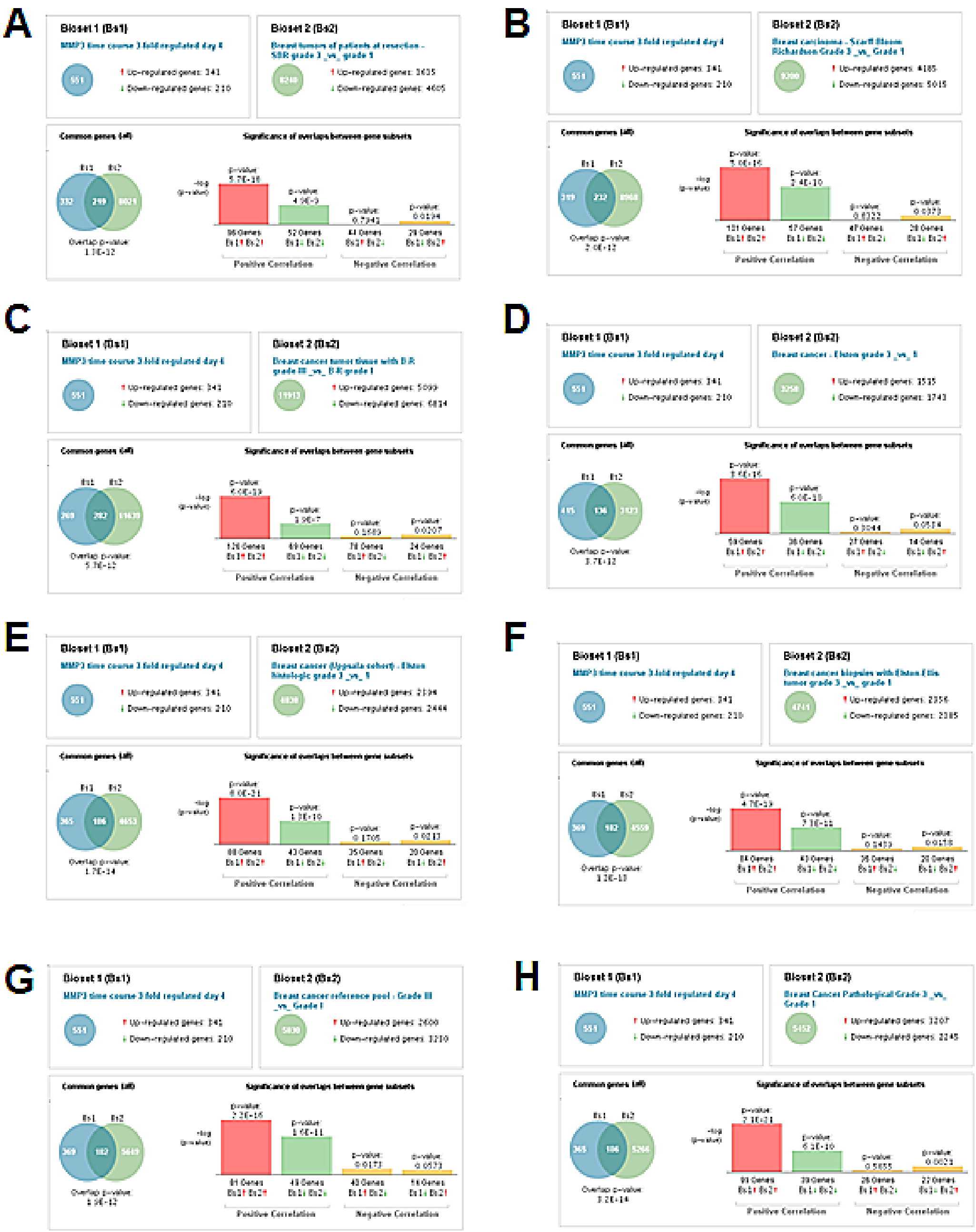

Supplemental Figure S1: Meta-analysis of datasets showing overlap of response to MMP-3 with biosets comparing breast cancer grade 3 to grade 1. In each panel, Bioset #1 is the 551 transcript list of response to MMP-3 (>3 FC on day 4). (A-C) Positive overlap with biosets comparing breast tumors with Scarff-Bloom-Richardson grade 3 vs grade 1 (A, [1] ; B, [2];C,

[3]). (D-F) Positive overlap with biosets comparing breast tumors with Elston grade 3 vs grade 1 (D, [4]; E, [5]; F, [6]). (G-H) Positive overlap with biosets comparing breast tumors with pathological grade 3 vs grade 1 (G, [7]; H, [8]).

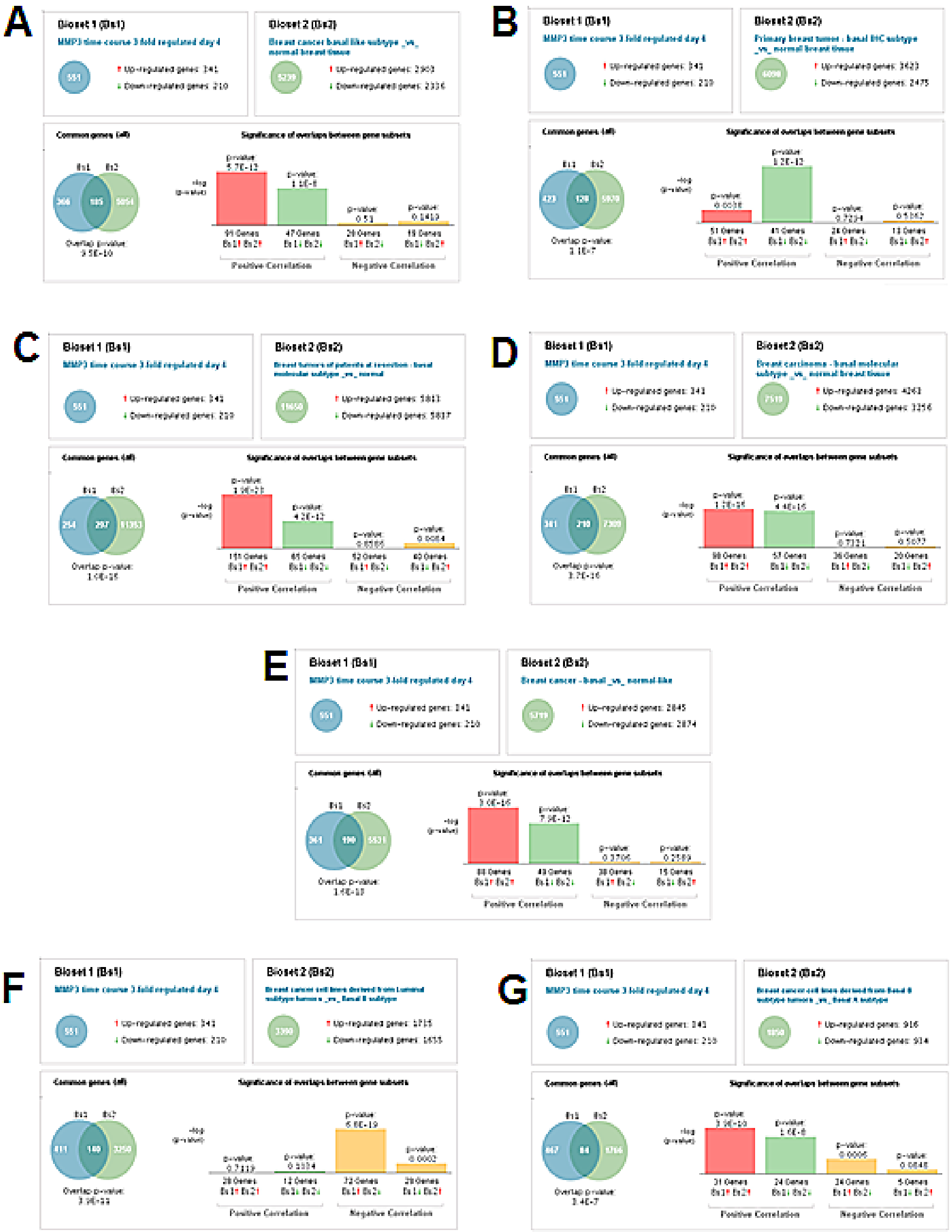

Supplemental Figure S2: Meta-analysis of datasets showing overlap of response to MMP-3 with biosets comparing breast cancer of basal subtype vs other subtypes. In each panel, Bioset #1 is the 551 transcript list of response to MMP-3 (>3 FC on day 4). (A-D) Positive overlap with biosets comparing breast tumors of the basal subtype vs normal breast tissue (A, [9]; B, [10]; C, [1]; D, [2]). (E) Positive overlap with bioset comparing breast tumors of basal subtype vs normal-like subtype [4]. (F) Negative overlap with bioset comparing breast cancer cell lines derived from luminal subtype vs Basal B subtype tumors [11]. (G) Positive overlap

with bioset comparing breast cancer cell lines derived from Basal B subtype tumors vs Basal A subtype tumors [11].

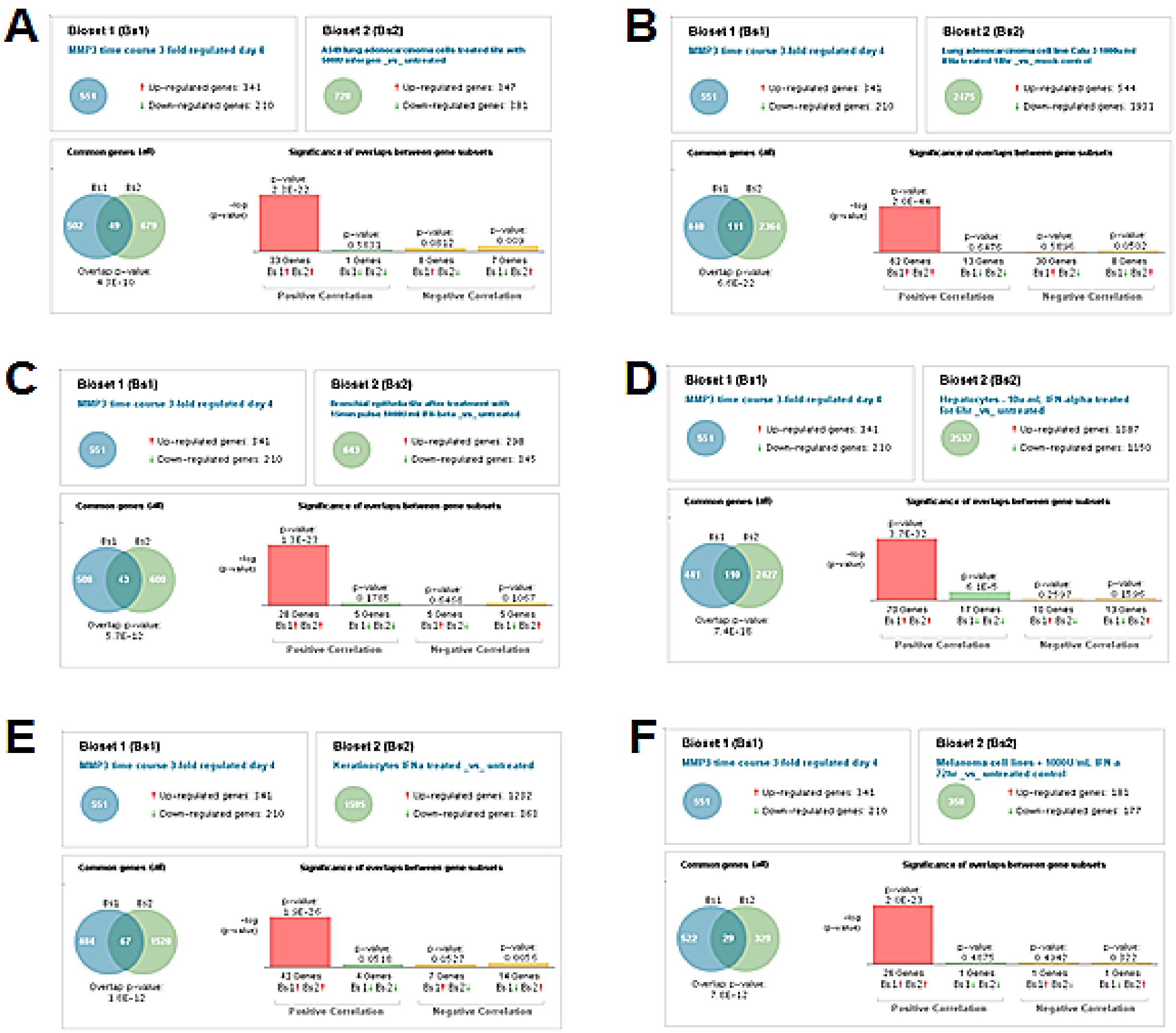

Supplemental Figure S3: Meta-analysis of datasets showing overlap of response to MMP-3 with biosets evaluating interferon response. In each panel, Bioset #1 is the 551 transcript list of response to MMP-3 (>3 FC on day 4). (A) Positive overlap with bioset comparing A549 lung cancer cells treated with infergen (IFN-αcon1) vs untreated [12]. (B) Positive overlap with bioset comparing Calu-3 lung cancer cells treated with IFN-α vs untreated [13]. (C) Positive overlap with bioset comparing primary human bronchial epithelial cells treated with IFN-β vs untreated [14]. (D) Positive overlap with bioset comparing JFH1 hepatocytes treated with IFN-β vs untreated [15]. (E) Positive overlap with bioset comparing human keratinocytes treated with IFN-α vs untreated [16]. (F) Positive overlap with bioset comparing human melanoma cell lines treated with IFN-α vs untreated [17].

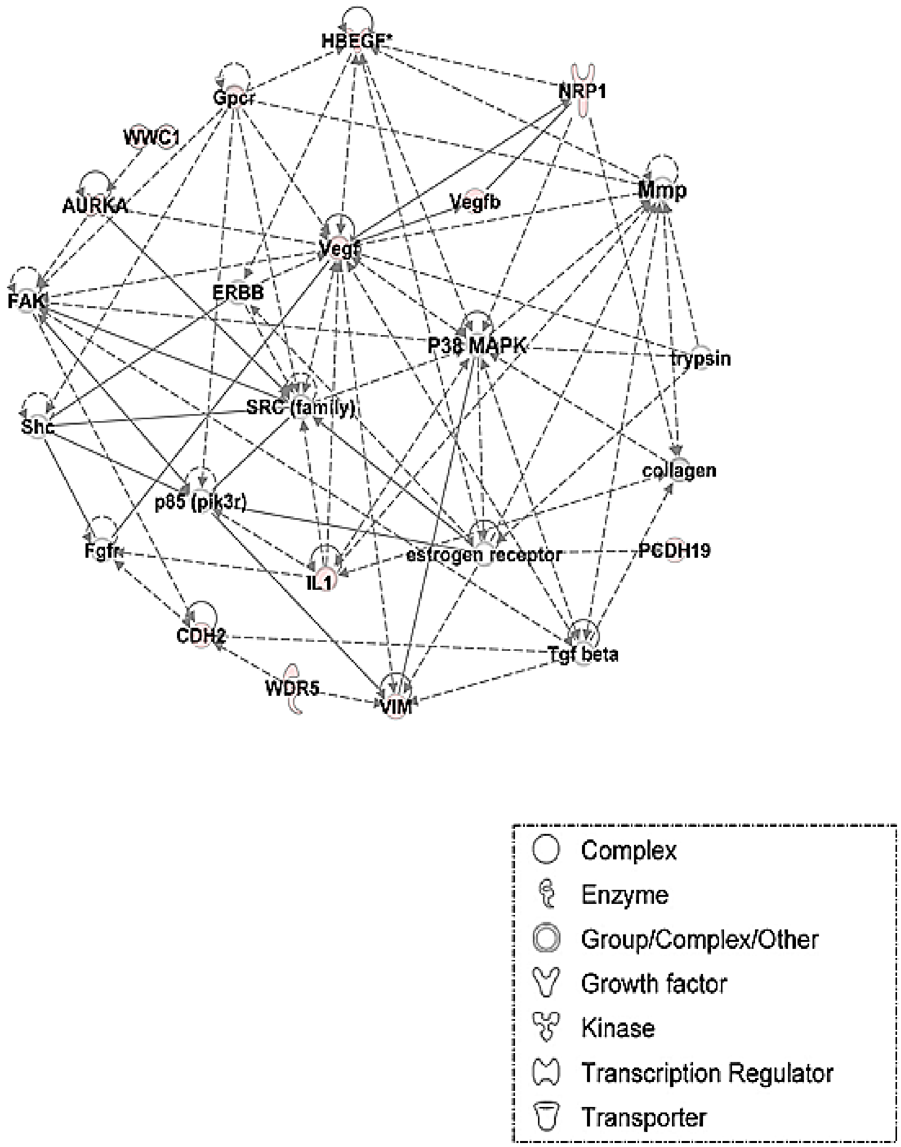

Supplemental Figure S4: IPA network containing effectors and regulators of epithelial-mesenchymal transition. IPA network of response to MMP-3 (intensity of red indicates degree of upregulation by MMP-3 on day 4) identified as containing regulators and effectors of EMT.

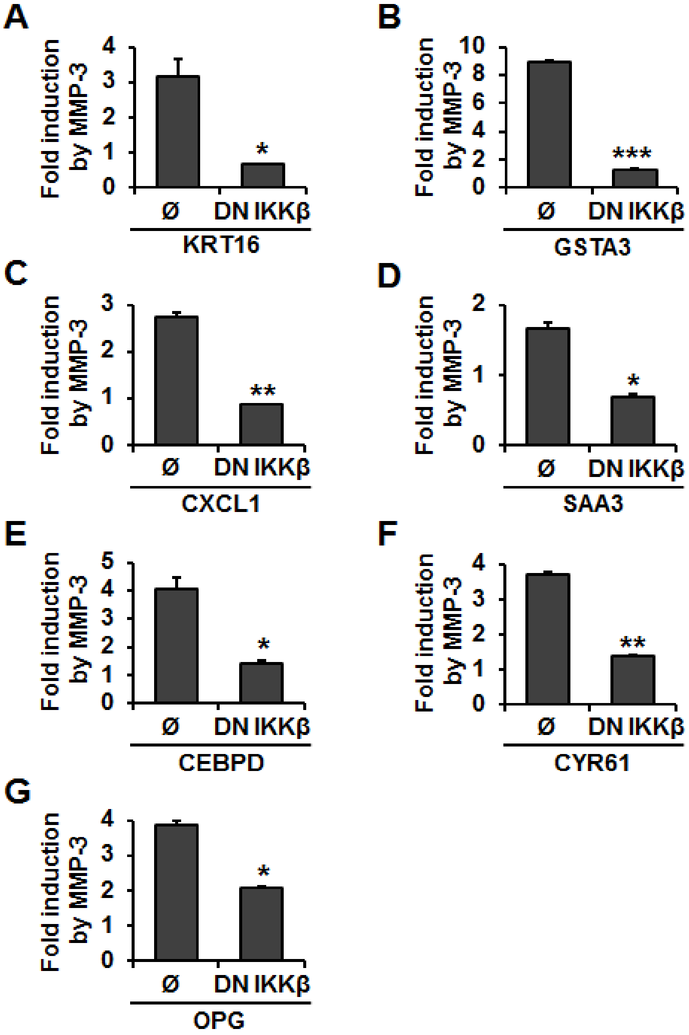

Supplemental Figure S5: Activation of MMP-3 responsive genes is NF-κB dependent. Cells were transfected with the indicated luciferase promoter reporters and co-transfected either as control or dominant-negative IKKβ. They were then exposed to MMP-3 and luciferase activity was measured. Graphs represent fold induction by MMP-3. Error bars, SE. (* $p<0.05$; ** $p<0.01$, *** $p<0.001$).

SUPPLEMENTAL FIGURE REFERENCES

1. Sircoulomb F, Bekhouche I, Finetti P, Adelaide J, Ben Hamida A, Bonansea J, Raynaud S, Innocenti C, Charafe-Jauffret E, Tarpin C, Ben Ayed F, Viens P, Jacquemier J, Bertucci F, Birnbaum D and Chaffanet M. Genome profiling of ERBB2-amplified breast cancers. BMC Cancer. 2010; 10:539.
2. Sabatier R, Finetti P, Adelaide J, Guille A, Borg JP, Chaffanet M, Lane L, Birnbaum D and Bertucci F. Down-regulation of ECRG4, a candidate tumor suppressor gene, in human breast cancer. PLoS One. 2011; 6(11):e27656.
3. Lu X, Wang ZC, Iglehart JD, Zhang X and Richardson AL. Predicting features of breast cancer with gene expression patterns. Breast Cancer Res Treat. 2008; 108(2):191-201.
4. Pawitan Y, Bjohle J, Amler L, Borg AL, Egyhazi S, Hall P, Han X, Holmberg L, Huang F, Klaar S, Liu ET, Miller L, Nordgren H, Ploner A, Sandelin K, Shaw PM, et al. Gene expression profiling spares early breast cancer patients from adjuvant therapy: derived and validated in two population-based cohorts. Breast Cancer Res. 2005; 7(6):R953-964.
5. Ivshina AV, George J, Senko O, Mow B, Putti TC, Smeds J, Lindahl T, Pawitan Y, Hall P, Nordgren H, Wong JE, Liu ET, Bergh J, Kuznetsov VA and Miller LD. Genetic reclassification of histologic grade delineates new clinical subtypes of breast cancer. Cancer Res. 2006; 66(21):10292-10301.
6. Miller LD, Smeds J, George J, Vega VB, Vergara L, Ploner A, Pawitan Y, Hall P, Klaar S, Liu ET and Bergh J. An expression signature for p53 status in human breast cancer predicts mutation status, transcriptional effects, and patient survival. Proc Natl Acad Sci U S A. 2005; 102(38):13550-13555.
7. Silver DP, Richardson AL, Eklund AC, Wang ZC, Szallasi Z, Li Q, Juul N, Leong CO, Calogrias D, Buraimoh A, Fatima A, Gelman RS, Ryan PD, Tung NM, De Nicolo A, Ganesan S, et al. Efficacy of neoadjuvant Cisplatin in triple-negative breast cancer. J Clin Oncol. 2010; 28(7):1145-1153.
8. Oncology) TIGCITepEPf. The International Genomics Consortium (IGC). The expO project (Expression Project for Oncology).
9. Parker JS, Mullins M, Cheang MC, Leung S, Voduc D, Vickery T, Davies S, Fauron C, He X, Hu Z, Quackenbush JF, Stijleman IJ, Palazzo J, Marron JS, Nobel AB, Mardis E, et al. Supervised risk predictor of breast cancer based on intrinsic subtypes. J Clin Oncol. 2009; 27(8):1160-1167.
10. Dedeurwaerder S, Desmedt C, Calonne E, Singhal SK, Haibe-Kains B, Defrance M, Michiels S, Volkmar M, Deplus R, Luciani J, Lallemand F, Larsimont D, Toussaint J, Haussy S, Rothe F, Rouas G, et al. DNA methylation profiling reveals a predominant immune component in breast cancers. EMBO Mol Med. 2011; 3(12):726-741.
11. Neve RM, Chin K, Fridlyand J, Yeh J, Baehner FL, Fevr T, Clark L, Bayani N, Coppe JP, Tong F, Speed T, Spellman PT, DeVries S, Lapuk A, Wang NJ, Kuo WL, et al. A collection of breast cancer cell lines for the study of functionally distinct cancer subtypes. Cancer Cell. 2006; 10(6):515-527.
12. Sanda C, Weitzel P, Tsukahara T, Schaley J, Edenberg HJ, Stephens MA, McClintick JN, Blatt LM, Li L, Brodsky L and Taylor MW. Differential gene induction by type I and type II interferons and their combination. J Interferon Cytokine Res. 2006; 26(7):462-472.

13. Li C, Bankhead A, 3rd, Eisfeld AJ, Hatta Y, Jeng S, Chang JH, Aicher LD, Proll S, Ellis AL, Law GL, Waters KM, Neumann G, Katze MG, McWeeney S and Kawaoka Y. Host regulatory network response to infection with highly pathogenic H5N1 avian influenza virus. J Virol. 2011; 85(21):10955-10967.
14. Shapira SD, Gat-Viks I, Shum BO, Dricot A, de Grace MM, Wu L, Gupta PB, Hao T, Silver SJ, Root DE, Hill DE, Regev A and Hacohen N. A physical and regulatory map of host-influenza interactions reveals pathways in H1N1 infection. Cell. 2009; 139(7):1255-1267.
15. Thomas E, Gonzalez VD, Li Q, Modi AA, Chen W, Noureddin M, Rotman Y and Liang TJ. HCV infection induces a unique hepatic innate immune response associated with robust production of type III interferons. Gastroenterology. 2012; 142(4):978-988.
16. Swindell WR, Xing X, Stuart PE, Chen CS, Aphale A, Nair RP, Voorhees JJ, Elder JT, Johnston A and Gudjonsson JE. Heterogeneity of inflammatory and cytokine networks in chronic plaque psoriasis. PLoS One. 2012; 7(3):e34594.
17. Kholmanskikh O, van Baren N, Brasseur F, Ottaviani S, Vanacker J, Arts N, van der Bruggen P, Coulie P and De Plaen E. Interleukins 1alpha and 1beta secreted by some melanoma cell lines strongly reduce expression of MITF-M and melanocyte differentiation antigens. Int J Cancer. 2010; 127(7):1625-1636.
